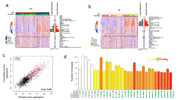# Spatial proteome profiling for distinguishing tangle‐bearing neurons and their microenvironments from healthy neurons in Alzheimer’s disease brains

**DOI:** 10.1002/alz.089552

**Published:** 2025-01-03

**Authors:** Shiva Kazempour Dehkordi, Timothy C. Orr, Ryan H Bodamer, Hannah R. Hudson, Victoria Sun, C Dirk Keene, Habil Zare, Miranda E. Orr

**Affiliations:** ^1^ University of Texas Health Science Center at San Antonio, San Antonio, TX USA; ^2^ Wake Forest University School of Medicine, Winston‐Salem, NC USA; ^3^ Department of Laboratory Medicine and Pathology, University of Washington, Seattle, WA USA; ^4^ The University of Texas Health Science Center at San Antonio, San Antonio, TX USA

## Abstract

**Background:**

An important hallmark of Alzheimer’s Disease (AD) is the presence of neurofibrillary tangles (NFTs) composed of phosphorylated tau, which are commonly assessed using AT8 immunostains. Identifying additional markers to characterize the spectrum of NFT pathology is crucial for advancing our understanding and diagnosis of AD. This study introduces new potential markers to differentiate between tangles and healthy neurons.

**Method:**

Postmortem human AD brains were analyzed using GeoMx (NanoString, Inc) digital spatial profiling. The analysis focused on specific regions of interest (ROIs) in the hippocampal CA1 and entorhinal cortex (EC) region of the brain, chosen for their presence or absence of AT8. We measured the levels of 85 proteins associated with neuropathology, inflammation, and autophagy across these ROIs. Differentially expressed (DE) proteins in NFTs versus healthy neurons were identified. The top 10 DE candidate proteins were used to construct an eigengene, a weighted average expression of proteins, to summarize biological signatures. We also trained a classifier using a non‐linear support vector machine to distinguish NFTs from healthy neurons, which is validated on an independent unlabeled dataset.

**Result:**

Tau phosphorylation at four specific epitopes, S199, S214, S396, and S404 were significantly elevated in tangle‐bearing neurons in both CA1 and EC (Fig. 1a‐b). Other top DE proteins, including P62, Neprilysin, Ubiquitin, and PSEN1, were also highlighted. Specifically, an eigengene combining these proteins exhibited distinct clusters separating AT8‐positive and negative cells (Fig. 1c). Our classification models using these markers as well as phosphorylated tau proteins showed significant concordance with AT8‐based labels, suggesting their potential as alternative diagnostic tools (Fig. 1d). Validating the association of each of these markers with tangles across the adult lifespan and with AD progression informs on the time course of their upregulation in relation to tau phosphorylation.

**Conclusion:**

This study identifies alternative markers, particularly phospho‐tau epitopes S214, S396, S404, and other proteins including P62, Neprilysin, and Ubiquitin for tangle‐bearing neurons. These markers are promising to improve AD diagnosis and fill gaps in knowledge regarding molecular pathogenesis of NFTs, paving the way for possibly earlier and more accurate detection and intervention strategies